# Impact of pulmonary valve replacement on left ventricular rotational mechanics in repaired tetralogy of Fallot

**DOI:** 10.1186/s12968-021-00750-3

**Published:** 2021-05-24

**Authors:** Jamie K. Harrington, Sunil Ghelani, Nikhil Thatte, Anne Marie Valente, Tal Geva, Julia A. Graf, Minmin Lu, Lynn A. Sleeper, Andrew J. Powell

**Affiliations:** 1 Department of Cardiology, Boston Children’s Hospital, Boston, MA USA; 2 Department of Pediatrics, Harvard Medical School, Boston, MA USA; 3 Department of Medicine, Brigham and Women’s Hospital, Boston, MA USA; 4 Department of Pediatrics, Division of Cardiology, College of Physicians and Surgeons, Columbia University, 3959 Broadway, CHN 2, New York, NY 10032 USA

**Keywords:** Feature tracking, Magnetic resonance imaging, Tetralogy of Fallot, Rotational mechanics, Twist, Torsion, Pulmonary valve replacement

## Abstract

**Background:**

In repaired tetralogy of Fallot (rTOF), abnormal left ventricular (LV) rotational mechanics are associated with adverse clinical outcomes. We performed a comprehensive analysis of LV rotational mechanics in rTOF patients using cardiac magnetic resonance (CMR) prior to and following surgical pulmonary valve replacement (PVR).

**Methods:**

In this single center retrospective study, we identified rTOF patients who (1) had both a CMR ≤ 1 year before PVR and ≤ 5 years after PVR, (2) had no other intervening procedure between CMRs, (3) had a body surface area > 1.0 m^2^ at CMR, and (4) had images suitable for feature tracking analysis. These subjects were matched to healthy age- and sex-matched control subjects. CMR feature tracking analysis was performed on a ventricular short-axis stack of balanced steady-state free precession images. Measurements included LV basal and apical rotation, twist, torsion, peak systolic rates of rotation and torsion, and timing of events. Associations with LV torsion were assessed.

**Results:**

A total of 60 rTOF patients (23.6 ± 7.9 years, 52% male) and 30 healthy control subjects (20.8 ± 3.1 years, 50% male) were included. Compared with healthy controls, rTOF patients had lower apical and basal rotation, twist, torsion, and systolic rotation rates, and these parameters peaked earlier in systole. The only parameters that were correlated with LV torsion were right ventricular (RV) end-systolic volume (r = − 0.28, p = 0.029) and RV ejection fraction (r = 0.26, p = 0.044). At a median of 1.0 year (IQR 0.5–1.7) following PVR, there was no significant change in LV rotational parameters versus pre-PVR despite reductions in RV volumes, RV mass, pulmonary regurgitation, and RV outflow tract obstruction.

**Conclusion:**

In this comprehensive study of CMR-derived LV rotational mechanics in rTOF patients, rotation, twist, and torsion were diminished compared to controls and did not improve at a median of 1 year after PVR despite favorable RV remodeling.

**Supplementary Information:**

The online version contains supplementary material available at 10.1186/s12968-021-00750-3.

## Background

Patients undergoing surgical repair of tetralogy of Fallot (rTOF) have excellent short-term survival [1–3]; however, they have an increased risk of heart failure, arrhythmia, and mortality later in life [4–7]. These sequelae are believed to be in part related to chronic pulmonary regurgitation (PR) and/or stenosis, and their adverse impact on the right heart. Thus, pulmonary valve replacement (PVR) is often performed in an effort to improve long-term outcomes [8–12].

Abnormal left ventricular (LV) function, however, is also seen in rTOF patients and is associated with adverse events [5, 6, 13–17]. Most studies examining this issue have focused on LV ejection fraction (LVEF) as the measure of function. Specifically, a depressed LVEF has been reported in 12–49% of rTOF patients [15–17], and has been associated with ventricular arrhythmias [5], impaired exercise intolerance [15], decreased event-free survival [17], and increased risk of sudden cardiac death [14]. Therefore, understanding the mechanism of LV dysfunction is crucial. More advanced measures of LV myocardial mechanics such as deformation, synchrony, and rotational mechanics may be more sensitive markers of LV dysfunction [18–23]. Rotational mechanics, such as twist and torsion, have been less well studied, but prior reports have indicated that these measures are abnormal in rTOF patients [20, 24–31], and are associated with adverse outcomes [21]. Most of these studies used echocardiography to measure rotational parameters which may be technically limited by reduced acoustic windows, particularly in post-operative patients. Moreover, a comprehensive understanding of the impact of PVR on LV rotational mechanics in rTOF patients remains largely unknown.

Therefore, we used cardiovascular magnetic resonance imaging (CMR) to perform a detailed analysis of LV rotational mechanics in rTOF patients before and after surgical PVR. A more thorough understanding of LV dysfunction and the effects of PVR on LV myocardial mechanics may lead to improved indications and outcomes for the procedure.

## Methods

### Subjects

In this single center study, rTOF subjects were retrospectively identified who met the following inclusion criteria: (1) had a CMR both ≤ 1 year before surgical PVR and ≤ 5 years after surgical PVR performed at Boston Children’s Hospital since January 1, 2002, (2) had no other intervening cardiac procedure between CMRs, (3) had predominantly volume loading (TOF-PR) or pressure loading (TOF-RVOTO) physiology at the time of PVR, (4) had a body surface area (BSA) > 1.0 m^2^ at the time of the first CMR, and (5) had CMR images suitable for feature tracking (FT) analysis. Patients with TOF-PR physiology were defined as having a PR fraction > 40% and a right ventricular (RV) outflow tract (RVOT) peak gradient by echocardiography ≤ 25 mm Hg and patients with TOF-RVOTO physiology were defined as having a PR fraction ≤ 30% and a RVOT peak gradient or TR peak gradient ≥ 40 mm Hg by echocardiography. Similar grouping criteria for rTOF patients have been used in prior publications [32–34]. These subgroups were predefined to permit an analysis of whether the impact of PVR on rotational mechanics differed between the two most common physiologies. Subjects were restricted to BSA > 1.0 m^2^ to avoid including a large range of heart sizes which could produce a confounding effect on torsion metrics and to analyze a cohort of patients undergoing PVR at the most common time-in late childhood and adulthood. Age and sex matched controls were identified who had a CMR at Boston Children’s Hospital interpreted as normal, and who had no systemic or genetic disease with cardiovascular impact. A statistical power analysis was performed for sample size estimation based on preliminary data showing that the standard deviation of the change in torsion after PVR was 0.44 deg/cm. Using a two-sided, α = 0.05 level one-sample test of the null hypothesis that the mean absolute change in torsion was 0, the projected sample size required to detect a mean change in torsion as small as 0.22 deg/cm (one-half standard deviation) with 80% power was 32 patients. Given that the standard deviation was based on preliminary data and in order to ensure adequate power, we aimed to have 60 rTOF subjects. These 60 subjects were randomly selected from patients meeting the inclusion criteria. We matched the rTOF subjects with 30 controls, a 2:1 matching strategy driven by the limited availability of age and sex matched control subjects who had undergone a CMR using the same technique as the rTOF subjects.

For the rTOF subjects, data were abstracted from the medical record and included age at initial TOF repair, type of TOF repair, total number of cardiac surgeries, age at PVR, total number of PVRs, age at CMR, electrocardiographic (ECG) QRS duration at the time of CMR, and the results of echocardiograms performed within 12 months of each CMR. The Committee for Clinical Investigation at Boston Children’s Hospital approved this study and waived the requirement for informed consent.

### Cardiovascular magnetic resonance

The CMR protocol used for patients with rTOF has been previously published [35]. Briefly, all CMRs were performed on a 1.5 T CMR scanner (Achieva, Phillips Healthcare, Best, the Netherlands) using surface coils selected based on patient size. Imaging included a 12–14 slice stack (slice thickness 8–10 mm) of breath-hold, ECG-gated, balanced steady-state free precession (bSSFP) cine acquisitions in the short-axis plane to completely cover both ventricles. Twenty images per cardiac cycle were acquired and reconstructed to 30 images per cardiac cycle. ECG-gated phase contrast flow measurements were performed in the main pulmonary artery. Ventricular volumes and blood flow were measured using commercially available software (cvi42, Circle Cardiovascular Imaging Inc., Calgary, Alberta, Canada; and QMass, Medis Medical Imaging Systems, Leiden, the Netherlands).

### Image analysis

**Fig. 1:**
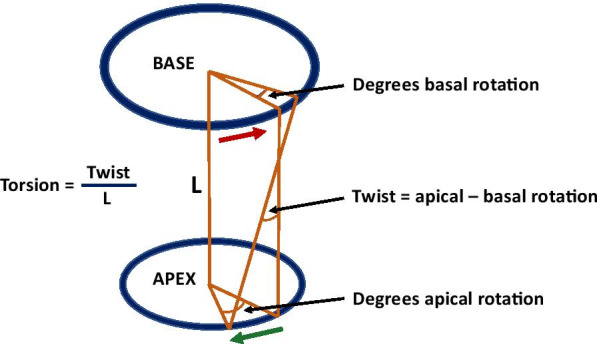
Schematic illustrating rotational mechanics. L is the length between the basal and apical slices

LV rotational mechanics were measured by performing FT analysis on the short-axis bSSFP cine images using commercially available software (cvi42, Circle Cardiovascular Imaging). FT is an image processing technology that quantifies myocardial tissue deformation [36]. LV endocardial and epicardial contours were manually drawn at end-systole for all slices from the apex to base of the LV and were propagated by the software through the remaining phases. In cases of poor tracking, the contours were manually adjusted. The apical slice was defined as the most apical slice with blood pool throughout the entire cardiac cycle. The basal slice was defined as the most basal slice with a full rim of myocardium throughout the entire cardiac cycle. The length of the LV was defined as the distance between the apical and basal slice. The following parameters were assessed: direction of rotation (clockwise or counterclockwise), degrees of apical and basal rotation, rate of peak systolic and diastolic apical and basal rotation, peak systolic twist (synchronous apical−basal rotation), peak systolic torsion, and peak systolic and diastolic rate of torsion (Fig. 1). Peak systolic torsion was calculated using 2 methods: (1) twist divided by LV length and (2) calculating the gradient of twist down the long-axis of the LV by finding the slope of the linear regression line between twist and longitudinal position [37]. End-systolic torsion was also calculated by the second method. As there was no significant difference in torsion between the methods, data is only presented for the peak systolic torsion calculated as the twist divided by LV length. The timing of all events was expressed as a percentage of the LV systolic duration. The beginning of systole was defined as phase 0 (0% of LV systolic duration) and end-systole was defined as the phase with the smallest LV volume (100% of LV systolic duration). Rotation was defined as positive if there was counterclockwise rotation when viewing the short-axis image from the apex. Intraobserver variability of the LV rotational parameters was assessed by repeated measurement of approximately 15% of the total studies by the primary observer (J.K.H). Analysis was performed 4 weeks after the primary assessment to limit recall bias. Interobserver variability was assessed in these same studies by a second observer (N.T.).

### Statistical analysis

Data are presented as mean ± standard deviation or median with interquartile range (IQR) as appropriate for continuous variables and as frequency (percentage) for categorical variables. A Student’s t-test was performed for mean comparisons between the rTOF and healthy control groups and between the rTOF patients with differing physiology. A Fisher exact test was performed for comparisons of categorical variables. Analysis of variance (ANOVA) was conducted for mean comparisons among 3 groups. If there was a significant 3-group difference, the p-value was adjusted for pairwise comparisons using the Tukey adjustment for multiple comparisons. The Benjamini–Hochberg false discovery rate method was applied to the total set of statistical tests performed for this study. A two-sided one sample t-test was used to assess whether the mean change in CMR and echocardiographic parameters between the first and second CMR was different from 0. The Pearson correlation coefficient was used to assess the correlation of clinical measures with peak torsion. Intra- and interobserver reproducibility were evaluated by estimation of the intraclass correlation coefficient (ICC). A p-value of ≤ 0.05 was considered statistically significant. Statistical analyses were performed using SAS (version 9.4, SAS Institute, Inc., Cary, North Carolina, USA) and R (version 3.2.1, R Foundation for Statistical Computing, Vienna, Austria).

## Results

### Subjects

There were 60 rTOF patients (23.6 ± 7.9 years, 52% male) and 30 controls (20.8 ± 3.1 years, 50% male). Their demographic and clinical information are presented in Table 1. At birth, 44 (73%) of the rTOF patients had pulmonary stenosis and 16 (27%) had pulmonary atresia. Additional anatomic features are listed in Table 1. The majority of the rTOF patients had a transannular patch repair (57%). The mean age at surgical repair was 2.1 ± 3.6 years. The median time between the pre-PVR CMR and PVR was 0.25 years (IQR: 3 days, 0.38 years), and the median time between the PVR and the post-PVR CMR was 1.00 years (IQR: 0.54, 1.74 years). There were significant differences in the type of TOF repair, number of surgeries, and number of PVRs between the TOF-PR and TOF-RVOTO subgroups (Table 1). The CMR indications for the control patients were suspected arrhythmogenic right ventricular cardiomyopathy (n = 18), family history of cardiomyopathy (n = 7), family history of a bicuspid aortic valve (n = 2), and other (n = 3). The patients with possible cardiomyopathy had a normal CMR as well as normal clinical and genetic evaluations.

**Table 1:** Subject characteristics

	TOF (n = 60)	Healthy control (n = 30)	P-value	TOF-PR (n = 38)	TOF-RVOTO (n = 22)	P-value^*^
Male, n (%)	31 (51.7)	15 (50.0)	1.00	19 (50.0)	12 (54.5)	0.793
Age at repair (years)	2.1 ± 3.6			1.1 ± 1.3	3.8 ± 5.4	**0.031**
Age at first CMR (years)	23.6 ± 7.9	20.8 ± 3.1	0.061	24.2 ± 6.9	22.5 ± 9.4	0.404
Age at second CMR (years)	25.2 ± 8.1			26.0 ± 7.0	23.7 ± 9.8	0.300
Age at PVR (years)	23.8 ± 7.9			24.6 ± 6.9	22.6 ± 9.4	0.345
Time between first CMR and PVR, median (IQR) (years)	0.25 (3 days, 0.38)			0.30 (0.10, 0.56)	0.01 (1d, 0.19)	** < 0.001**
Time between PVR and second CMR, median (IQR) (years)	1.00 (0.54, 1.74)			1.06 (0.67, 7.19)	0.57 (0.51, 1.69)	0.078
*Native anatomy*						
TOF-Pulmonary stenosis	44 (73.3)					
TOF-Pulmonary atresia	16 (26.7)					
*Additional anatomic features*						
Absent pulmonary valve	3 (5.0)					
Absent left pulmonary artery	3 (5.0)					
Discontinuous pulmonary arteries	4 (6.7)					
Atrioventricular canal defect	1 (1.7)					
*Initial repair, n (%)*						
Staged	18 (30)					
Complete	42 (70)					
*Type of complete repair, n (%)*						** < 0.001**
Transannular patch	34 (56.7)			30 (78.9)	4 (18.2)	
RV-PA conduit	15 (25.0)			0 (0)	15 (68.2)	
Non-Transannular patch	10 (16.7)			7 (18.4)	3 (13.6)	
Unknown	1 (1.7)			1 (2.6)	0 (0)	
*Number of surgeries, n (%)*						** < 0.001**
1	49 (81.7)			37 (97.4)	12 (54.5)	
2	8 (13.3)			1 (2.6)	7 (31.8)	
≥ 3	3 (5.0)			0 (0)	3 (13.6)	
*First PVR, n (%)*						** < 0.001**
Yes	51 (85.0)			38 (100)	13 (59.1)	
No	9 (15.0)			0 (0)	9 (40.9)	

Bold values are statistically significant p-values

The TOF-PR subgroup is defined as repaired TOF patients with a pulmonary regurgitation fraction > 40% and a right ventricular outflow tract peak gradient ≤ 25 mm Hg by echocardiography. The TOF-RVOTO subgroup is defined as repaired TOF patients with a PR fraction ≤ 30%, and a RVOT peak gradient or tricuspid regurgitation peak gradient ≥ 40 mm Hg by echocardiography. Values are mean ± standard deviation, median (interquartile range), or N (%). P-value^*^ refers to the comparison between the TOF-PR and TOF-RVOTO subgroups. CMR, cardiovascular magnetic resonance; IQR, interquartile range; PVR, pulmonary valve replacement; RV-PA, right ventricle-to-pulmonary artery; TOF, tetralogy of Fallot

### Conventional cardiovascular magnetic resonance and echocardiographic parameters

The conventional CMR and echocardiographic parameters for the rTOF patients and the healthy controls are presented in Table 2. The rTOF patients had larger RV volumes and mass and lower RV ejection fraction (RVEF) and LVEF on their pre-PVR CMR compared with healthy controls. After PVR in the rTOF patients, RV volumes, RV mass, PR fraction, and RVOT peak gradient significantly decreased. There was not a significant change in the RVEF or LVEF after PVR.

**Table 2:** Conventional cardiovascular magnetic resonance and echocardiographic parameters

	TOF Pre-PVR (n = 60)	TOF Post-PVR (n = 60)	Controls (n = 30)	TOF Pre-PVR vs. controls P-value	TOF Post-PVR vs. controls P-value	TOF Pre-PVR vs. TOF Post-PVR P-value
Heart rate (bpm)	73.6 ± 14.2	69.4 ± 13.5	73.9 ± 16.6	0.925	0.165	**0.006**
Height (cm)	164.3 ± 11.1	164.4 ± 10.7	169.9 ± 7.6	**0.006**	**0.006**	0.725
Weight (kg)	64.7 ± 17.9	67.4 ± 18.4	75.1 ± 19.1	**0.013**	0.070	** < 0.001**
BSA (m^2^)	1.7 ± 0.3	1.8 ± 0.3	1.9 ± 0.3	**0.007**	**0.038**	** < 0.001**
RVEDVI (ml/m^2^)	181.7 ± 55.6	128.0 ± 54.1	92.5 ± 13.1	** < 0.001**	** < 0.001**	** < 0.001**
RVESVI (ml/m^2^)	102.3 ± 48.4	75.6 ± 50.1	39.2 ± 7.6	** < 0.001**	** < 0.001**	** < 0.001**
RV mass index (g/m^2^)	37.7 ± 14.9	29.4 ± 12.5	17.3 ± 5.3	** < 0.001**	** < 0.001**	** < 0.001**
LVEDVI (ml/m^2^)	87.2 ± 19.0	92.5 ± 15.1	88.8 ± 11.9	0.621	0.246	**0.009**
LVESVI (ml/m^2^)	39.5 ± 13.3	40.7 ± 10.6	34.3 ± 6.4	**0.014**	** < 0.001**	0.296
LV mass index (g/m^2^)	53.6 ± 12.2	54.4 ± 11.7	53.1 ± 12.3	0.857	0.639	0.606
RVEF (%)	44.9 ± 9.8	43.7 ± 8.9	57.8 ± 4.6	** < 0.001**	** < 0.001**	0.220
LVEF (%)	55.3 ± 7.3	56.5 ± 5.9	61.4 ± 5.0	** < 0.001**	** < 0.001**	0.094
PR (%)	40 ± 20	6 ± 11				** < 0.001**
QRS (ms)	149 ± 24	146 ± 26				0.054
RVOT PG (mm Hg)	28 ± 23	21 ± 13				**0.021**
TR PG (mm Hg)	47 ± 24	39 ± 22				**0.013**
*TR severity; n (%)*						
None/trivial	15 (27.3)	19 (33.9)				
Mild	31 (56.4)	32 (57.1)				
Moderate	8 (14.5)	4 (7.1)				
Severe	1 (1.8)	1 (1.8)				

Bold values are statistically significant p-values

Values are mean ± standard deviation or N (%). BSA, body surface area; LV, left ventricular; LVEDVI, left ventricular end-diastolic volume index; LVEF, left ventricular ejection fraction; LVESVI, left ventricular end-systolic volume index; PG, peak gradient; PR, pulmonary regurgitation; RV, right ventricular; RVEDVI, right ventricular end-diastolic volume index; RVEF, right ventricular ejection fraction; RVESVI, right ventricular end-systolic volume index; RVOT, right ventricular outflow tract; TOF, tetralogy of Fallot; TR, tricuspid regurgitation

### Rotational mechanics

**Fig. 2:**
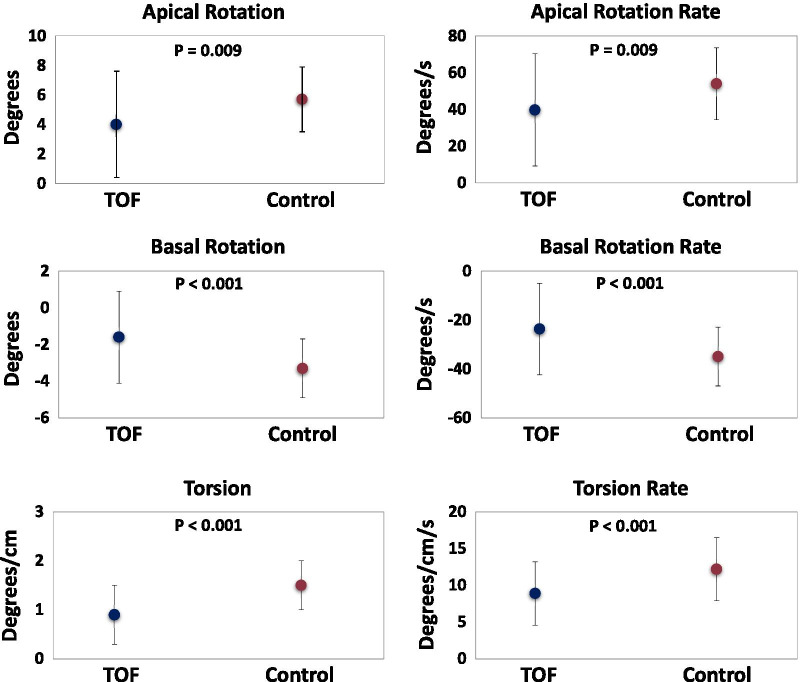
Peak systolic rotational parameters in repaired tetralogy of Fallot (rTOF) patients prior to pulmonary valve replacement (n = 60) compared to controls (n = 30). The circles represent the mean rotational parameters with standard deviation

**Fig. 3:**
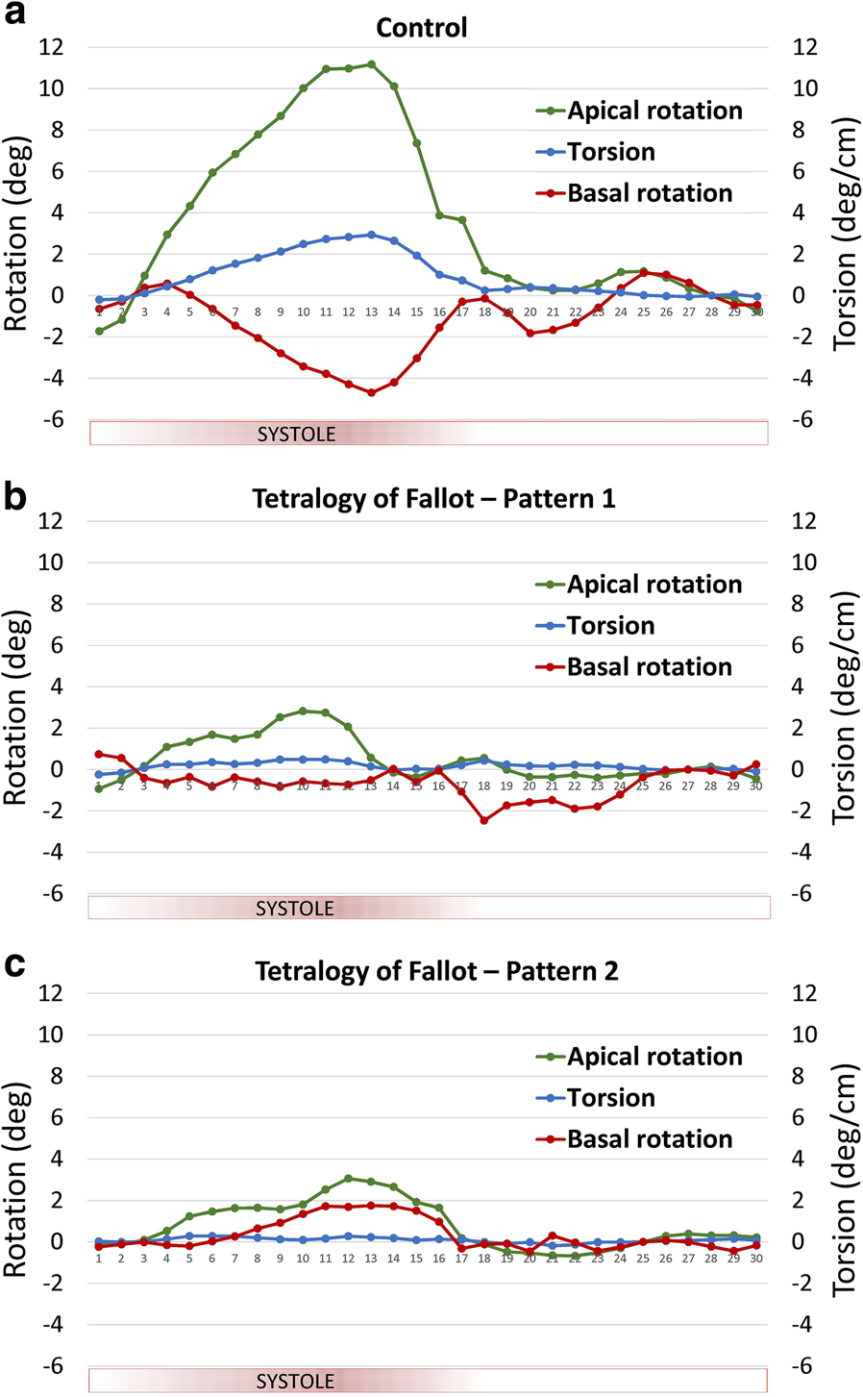
Rotational parameters over the cardiac cycle. **a** A representative control subject with counterclockwise apical rotation and clockwise basal rotation. **b** A representative repaired tetralogy of Fallot patient with the most common pattern of reduced apical and basal rotation with normal rotation direction. **c** A representative repaired tetralogy of Fallot patient with the second most common pattern of reversed basal rotation and normal direction but reduced apical rotation

The rotational mechanics from the CMR FT analysis for the rTOF patients and healthy controls are presented in Table 3 and Fig. 2 (and Additional file 1: Table S1). Compared with healthy controls, pre-PVR rTOF patients had significantly lower apical and basal rotation, twist, torsion, and systolic rotation rates, and apical rotation and torsion peaked earlier in systole. All of the healthy control patients had counterclockwise apical rotation and clockwise basal rotation, a normal pattern reported by others [38, 39]. There were two predominant rotational patterns seen in the rTOF cohort (Fig. 3). The majority of the rTOF patients (58%) had counterclockwise apical rotation and clockwise basal rotation (normal directions), but frequently the degree of rotation of the base and/or apex was reduced (Fig. 3b). The second most common pattern in the rTOF patients (32%) was reversed basal rotation and normal apical rotation direction (Fig. 3c).

**Table 3:** Left ventricular rotational mechanical parameters

	TOF Pre-PVR (n = 60)	TOF Post-PVR (n = 60)	Healthy controls (n = 30)	TOF Pre-PVR vs. controls P-value	TOF post-PVR vs. controls P-value	TOF Pre-PVR vs. TOF Post-PVR P-value
*Direction of rotation*						
Apical rotation counterclockwise, n (%)				0.173	0.548	
Yes	54 (90.0)	57 (95.0)	30 (100)			
No	6 (10.0)	3 (5.0)	0 (0)			
Basal rotation clockwise, n (%)				** < 0.001**	**0.004**	
Yes	40 (66.7)	46 (76.7)	30 (100)			
No	20 (33.3)	14 (23.3)	0 (0)			
Torsion positive, n (%)				0.297	0.548	
Yes	56 (93.3)	57 (95.0)	30 (100)			
No	4 (6.7)	3 (5.0)	0 (0)			
*Rotation degrees*						
Peak apical rotation (deg)	4.0 ± 3.6	4.5 ± 3.3	5.7 ± 2.2	**0.009**	**0.041**	0.380
Peak basal rotation (deg)	− 1.6 ± 2.5	− 1.2 ± 1.8	− 3.3 ± 1.6	** < 0.001**	** < 0.001**	0.203
Twist (deg)	5.9 ± 4.6	6.1 ± 4.4	10.0 ± 3.3	** < 0.001**	** < 0.001**	0.768
Torsion (deg/cm)	0.9 ± 0.6	1.0 ± 0.7	1.5 ± 0.5	** < 0.001**	** < 0.001**	0.443

Bold values are statistically significant p-values

Values are mean ± standard deviation or N (%). Deg, degrees; TOF, tetralogy of Fallot; and PVR, pulmonary valve replacement

**Fig. 4:**
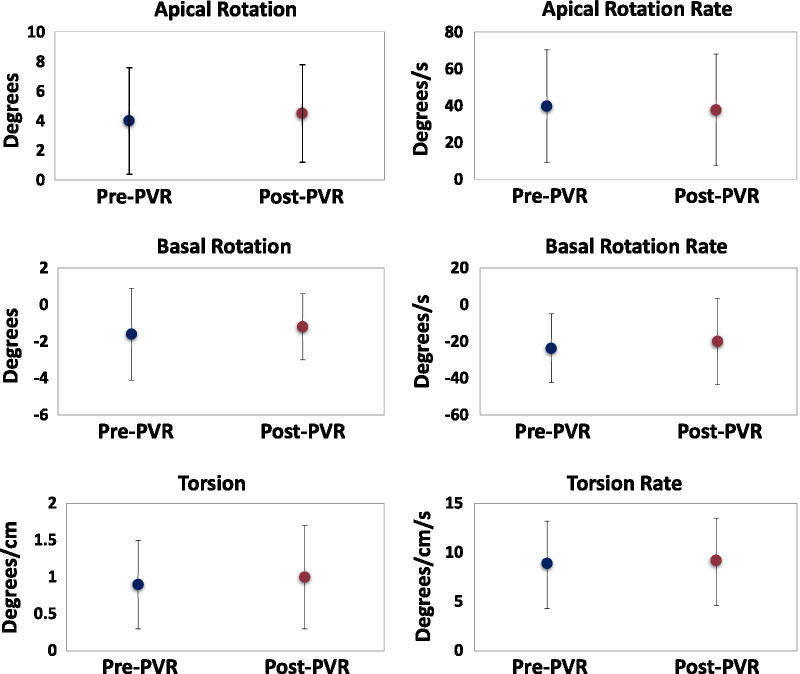
Peak systolic rotational parameters in repaired tetralogy of Fallot patients pre- and post-pulmonary valve replacement (n = 60). The circles represent the mean rotational parameters with standard deviations. P > 0.05 for all

In the rTOF patients, nearly all of the LV rotational parameters had no significant change following PVR (Table 3 and Fig. 4; and Additional file 1: Table S1). Of the 60 patients, only 16 patients (27%) showed an increase in torsion of > 0.5 deg/cm. The only rotational parameter that showed a significant change after PVR was the timing of peak torsion (Additional file 1: Table S1). After PVR, torsion peaked later in systole in the rTOF patients, closer to controls. After PVR, the number of patients with normal apical and basal rotation direction increased from 58 to 73%.

Two subgroups of rTOF patients were compared: patients with predominantly volume-loaded physiology (TOF-PR) (n = 38) and patients with predominantly pressure-loaded physiology (TOF-RVOTO) (n = 22) at the time of PVR (Table 4 and Additional file 1: Table S2). On the pre-PVR CMR, there were no significant differences between the two groups in the apical or basal rotation, twist, torsion, or rotation rates. However, apical rotation, apical systolic rotation rate, and torsion peaks occurred later in systole in the TOF-PR group. In both groups following PVR, there were no significant changes in the apical rotation, basal rotation, twist, or torsion.

**Table 4:** Subgroup analysis of repaired tetralogy of Fallot patients

Variable	Pre-PVR			Post-PVR			Pre-PVR vs. Post-PVR	
	TOF-PR (n = 38)	TOF-RVOTO (n = 22)	P-value	TOF-PR (n = 38)	TOF-RVOTO (n = 22)	P-value	TOF-PR P-value	TOF-RVOTO P-value
Heart rate (bpm)	69.4 ± 11.3	80.8 ± 16.0	**0.002**	67.6 ± 12.1	72.5 ± 15.3	0.173	0.153	**0.019**
Height (cm)	165.6 ± 11.8	162.0 ± 9.5	0.225	165.6 ± 11.5	162.4 ± 9.0	0.266	1.00	0.675
Weight (kg)	67.2 ± 18.8	60.4 ± 15.7	0.156	70.5 ± 19.9	62.1 ± 14.2	0.089	0.001	**0.045**
BSA (m^2^)	1.8 ± 0.3	1.6 ± 0.3	0.138	1.8 ± 0.3	1.7 ± 0.2	0.077	** < 0.001**	0.053
RVEDVI (ml/m^2^)	191.4 ± 29.0	164.8 ± 82.0	0.153	119.1 ± 28.8	143.2 ± 79.8	0.186	** < 0.001**	**0.003**
RVESVI (ml/m^2^)	98.0 ± 21.7	109.8 ± 75.2	0.478	65.7 ± 23.4	92.7 ± 75.0	0.114	** < 0.001**	**0.002**
RV mass index (g/m^2^)	34.8 ± 9.4	43.0 ± 20.8	0.098	26.3 ± 7.4	34.6 ± 17.2	**0.041**	** < 0.001**	** < 0.001**
LVEDVI (ml/m^2^)	84.2 ± 14.2	92.3 ± 24.7	0.168	90.8 ± 15.0	95.5 ± 15.3	0.241	** < 0.001**	0.514
LVESVI (ml/m^2^)	36.1 ± 8.3	45.3 ± 17.8	**0.031**	38.7 ± 9.2	44.3 ± 11.9	**0.045**	**0.032**	0.690
LV mass index (g/m^2^)	51.2 ± 8.9	57.8 ± 15.8	0.085	52.6 ± 10.9	57.5 ± 12.7	0.118	0.336	0.932
RVEF (%)	49.1 ± 5.8	37.5 ± 11.1	** < 0.001**	45.9 ± 6.5	40.0 ± 11.2	**0.033**	**0.001**	0.170
LVEF (%)	57.3 ± 5.4	52.0 ± 8.9	**0.016**	57.8 ± 4.6	54.1 ± 7.2	**0.036**	0.501	0.077
PR (%)	54 ± 8	17 ± 11	** < 0.001**	8 ± 13	2 ± 4	**0.007**	** < 0.001**	** < 0.001**
QRS (ms)	149 ± 24	150 ± 24	0.824	144 ± 24	150 ± 28	0.377	**0.026**	0.915
RVOT PG (mm Hg)	13 ± 8	52 ± 19	** < 0.001**	17 ± 6	27 ± 19	**0.040**	**0.018**	** < 0.001**
TR PG (mm Hg)	29 ± 8	69 ± 17	** < 0.001**	27 ± 9	53 ± 24	** < 0.001**	0.198	**0.027**
Peak apical rotation (deg)	4.7 ± 3.8	2.8 ± 3.0	0.054	5.0 ± 3.1	3.5 ± 3.4	0.089	0.652	0.372
Peak basal rotation (deg)	− 1.6 ± 2.9	− 1.7 ± 1.8	0.829	− 1.1 ± 1.7	− 1.3 ± 2.0	0.647	0.316	0.438
Twist (deg)	6.7 ± 5.0	4.7 ± 3.4	0.105	6.6 ± 4.0	5.3 ± 5.1	0.271	0.970	0.509
Torsion (deg/cm)	1.0 ± 0.7	0.8 ± 0.5	0.252	1.0 ± 0.6	0.9 ± 0.9	0.709	0.779	0.363

Bold values are statistically significant p-values

Values are mean ± standard deviation. TOF-PR are repaired tetralogy of Fallot (rTOF) patients with a pulmonary regurgitation fraction > 40% and a right ventricular outflow tract peak gradient ≤ 25 mm Hg. TOF-RVOTO are rTOF patients with a PR fraction ≤ 30% and a RVOT peak gradient or tricuspid regurgitation peak gradient ≥ 40 mm Hg. BSA, body surface area; Deg, degrees; rTOF, repaired tetralogy of Fallot; LVEDVi, left-ventricular end-diastolic volume index; LVESVI, left ventricular end-systolic volume index; LVEF, left ventricular ejection fraction; RVESVi, right ventricular end-systolic volume index; RVEF, right ventriuclar ejection fraction; RVESVI, right ventricular end-systolic volume index; PR, pulmonary regurgitation; PVR, pulmonary valve replacement; RV, right ventricular; RVOT PG, right ventricular outflow tract peak gradient; TOF, tetralogy of Fallot; TR, tricuspid regurgitation; TR PG, tricuspid regurgitation peak gradient

Additional analyses focused on torsion in the rTOF patients at the time of their pre-PVR CMR. There was no significant difference between the 34 patients repaired at < 1 year of age and the 26 patients repaired at ≥ 1 year of age (1.01 ± 0.63 versus 0.75 ± 0.66, p = 0.13), between the 18 patients with a staged repair and the 42 patients with a primary complete repair (0.80 ± 0.76 versus 0.94 ± 0.60, p = 0.44), or between the 31 patients repaired before 1990 and the 29 patients repaired in or after 1990 (0.89 ± 0.49 versus 0.91 ± 0.80, p = 0.94).

The intra- and interobserver reproducibility (n = 15) for peak systolic torsion as measured by the ICC were good (0.80, 95% CI 0.50, 0.93) and moderate (0.65, 95% CI 0.25, 0.87), respectively.

### Correlation with peak torsion

Table 5 reports the correlation between peak systolic torsion prior to PVR and clinical, CMR, and echocardiographic parameters in the rTOF group. Among all of these, only a weak correlation of peak torsion with RV end-systolic volume index (RVESVI) and with RVEF was detected. Late gadolinium enhancement of the LV and/or RV myocardium, beyond the common enhancement at the septal insertions, anterior infundibular free wall, and ventricular septal defect patch, was rare in this cohort (4 pre-PVR CMR studies) and was not included in the correlation analysis.

**Table 5:** Correlation with peak systolic torsion in repaired tetralogy of Fallot (n = 60)

	r	P-value
Age at CMR (years)	0.06	0.635
Gender		0.937
Age at TOF repair (years)	0.04	0.752
Type of TOF repair		0.444
Number of surgeries		0.290
Age at PVR (years)	0.06	0.629
Heart rate (bpm)	0.04	0.746
Height (cm)	0.13	0.311
Weight (kg)	0.13	0.308
BSA (m^2^)	0.14	0.281
RVEDVI (ml/m^2^)	− 0.19	0.136
RVESVI (ml/m^2^)	− 0.28	**0.029**
RV mass index (g/m^2^)	− 0.18	0.182
LVEDVI (ml/m^2^)	− 0.07	0.613
LVESVI (ml/m^2^)	− 0.13	0.310
LV mass index (g/m^2^)	− 0.08	0.520
PR (%)	0.12	0.343
RVEF (%)	0.26	**0.044**
LV F (%)	0.16	0.225
QRS (ms)	− 0.06	0.665
RVOT PG (mm Hg)	− 0.07	0.625
TR PG (mm Hg)	− 0.19	0.244

Bold values are statistically significant p-values

R is the Pearson correlation coefficient. BSA, body surface area; CMR, cardiovascular magnetic resonance; Deg, degrees; LVEDVI, left ventricular end-diastolic volume index; LVEF, left ventricular ejection fraction; LVESVI, indexed end-systolic volume; LV, left ventricular; PR, pulmonary regurgitation; PVR, pulmonary valve replacement; RV, right ventricular; RVEDVI, right ventricular end-diastolic volume index; RVEF, right ventricular ejection fraction; RVESVI, right ventricular end-systolic volume index; RVOT PG, right ventricular outflow tract peak gradient; TOF, tetralogy of Fallot; TR, tricuspid regurgitation; TR PG, tricuspid regurgitation peak gradient

## Discussion

In this comprehensive analysis of CMR-derived LV rotational mechanics in rTOF patients before and after surgical PVR, we found that compared to heathy control patients, rTOF patients had lower apical and basal rotation, twist, torsion, and systolic rotation rates. Nearly half of the rTOF patients demonstrated reversed basal and/or apical rotation. Among a variety of clinical, CMR, and echocardiographic parameters, torsion correlated only with RVESVI and with RVEF. At a median of 1 year after PVR, despite reductions in RV volumes, RV mass, PR, and RVOT obstruction, there was no improvement in peak apical or basal rotation, twist, torsion, or rotation rates. The overall finding of reduced torsion and twist pre-PVR with minimal change post-PVR was also seen in both the predominantly volume-loaded subgroup and the predominantly pressure-loaded subgroup. These findings provide detailed insight into the impaired LV rotation mechanics in rTOF patients.

Impaired LV function has been shown to be an important predictor of adverse clinical outcome in rTOF [6, 14]; however, the mechanism for LV dysfunction has not been well characterized. There has been an increasing interest on using advanced non-invasive measures of LV myocardial mechanics to gain insight into the underlying causes of LV dysfunction [21, 22, 26, 40]. The focus has primarily been on strain parameters, but rotation is also an essential part of the normal mechanics of the LV. The opposing rotation of the base and apex creates a wringing or twisting motion that is critical to optimal LV ejection [41]. While most of the rTOF patients in our study had preserved opposing clockwise basal rotation and counterclockwise apical rotation, the magnitude of rotation was significantly reduced compared to controls leading to a smaller twisting motion of the heart and reduced torsion. A smaller, but not insignificant, subset had more profound abnormalities with reversal of the normal rotational direction at either the base or apex eliminating the wringing motion.

It has been proposed that the LV rotational abnormalities in rTOF are related to unfavorable ventricular-ventricular interactions [6, 20, 24, 42–44]. The RV and LV epicardial fibers are continuous [45], and therefore, RV dilation, hypertension, hypertrophy, and dysfunction can lead to stretching or impaired motion of these fibers which may impede LV rotation. Moreover, the ventricles can influence each other through the shared septal wall. Our results, however, do not support the notion that ventricular-ventricular interactions play an important role in the LV rotational abnormalities based on data at a median of 1 year after PVR. Specifically, we did not find a substantial difference in LV rotation between rTOF patients with volume-loaded versus pressure-loaded RV physiology, nor identify strong correlations between RV parameters and LV torsion. Moreover, despite significant reductions in RV volumes, RV mass, PR, and RVOT obstruction after PVR, there were no significant changes in the LV rotational parameters at a median follow-up of 1 year. Thus, alternative causes for LV rotational dysfunction should be considered including abnormal electrical activation, congenital or acquired malformations in fiber orientation, and acquired pathological changes at the tissue level (e.g., fibrosis) [46]. In our cohort positive myocardial enhancement on late gadolinium enhancement imaging was rare, and T1 and extracellular volume mapping were not routinely performed, however, this may be of interest for future studies to investigate the impact of fibrosis on LV myocardial mechanics.

Our findings support and expand upon a small number of previously published works, mostly using speckle-tracking echocardiography [20, 21, 24–31, 47] and a couple using CMR [23, 48], that have shown that rotational mechanics are impaired in rTOF. Several studies found that abnormalities in basal rotation were predominantly driving the impairment in torsion [20, 23, 26, 29]. This may result from the RVOT and superior portions of the RV being more dilated and dysfunctional than the apex thereby resulting in more negative effects on the base of the LV [23]. However, even among these studies, RV end-diastolic volume index (RVEDVI) has not been consistently shown to be a significant predictor of abnormal basal rotation. In fact, Dragulescu et al. found that only RV longitudinal strain, and not RVEDVI, was a significant predictor of counterclockwise basal rotation, suggesting that RV function may be a more important determinant of abnormal LV rotation than RV dilation [26]. Other reports have documented more pronounced abnormalities in apical rotation [25, 30]. Yamada et al. proposed a time course for LV dysfunction in rTOF using layer specific strain where they found myocardial impairment originating in the endocardium at the base of the LV and extending to the apex from the endocardium to the epicardium [49]. Based on this theory and an understanding that apical rotation is driven by fibers near the epicardium, it can be hypothesized that abnormal apical rotation may represent more advanced disease. In our study, only 6 rTOF patients had reversed clockwise apical rotation which may indicate that our cohort had less advanced disease. Recently, van Grootel et al. showed that LV apical rotation assessed by speckle-tracking echocardiography was independently associated with a composite of death, heart failure, arrhythmia, reintervention, or hospitalization for cardiac reasons and recommended LV rotation as a routine part of assessment in rTOF patients [21]. More research is necessary to understand why abnormal apical rotation develops and how it may be prevented or improved.

There have also been a few studies that have similarly shown that various rotational parameters do not significantly change after PVR [20, 24, 25, 50]. Yim et al. found that post-PVR speckle-tracking echocardiography-derived apical and basal LV rotation values were not significantly different from pre-PVR values [20]. Menting et al. similarly found that speckle tracking echocardiography-derived apical and basal rotation and twist values were similar between rTOF without PVR, recent PVR, and PVR > 1 year before evaluation [25]. One small study using FT CMR found that LV torsion remained unchanged after PVR in 10 rTOF patients [50]. Our study confirms and expands upon these findings by adding a more comprehensive assessment of rotational mechanics both pre- and post-PVR in a large cohort of rTOF patients by FT CMR.

Other CMR methods have been used to assess LV rotational mechanics in rTOF patients. A recent study using tissue phase mapping found altered twist function in 47 rTOF patients with preserved LVEF [48]. Similarly, Ordovas et al. found delayed LV peak rotation in rTOF patients with preserved LVEF using CMR tissue tagging [23]. Although some studies have suggested that FT has inferior reproducibility and suboptimal agreement with other CMR methods for assessing myocardial deformation [37, 51], other studies have shown acceptable correlation between FT and methods such as myocardial tagging [52, 53]. In addition, FT has the advantage of requiring only conventional cine sequences which are a routine part of clinical studies rather than an additional acquisition.

## Limitations

Our study has several limitations. As no difference in torsion was found following PVR, an analysis of statistical power is of interest. Using the observed (rather than preliminary estimate) standard deviation of the mean change in torsion following PVR (0.84 deg/cm), with 60 patients, there was 80% power to detect a mean change of 0.3 deg/cm in torsion using a two-sided 0.05 level test. Differences less than 0.3 deg/cm seem unlikely to be clinically significant. Our study included only patients who had undergone surgical PVR; the impact of transcatheter PVR on rotational parameters was not assessed. Post-PVR assessment was performed at a median of 1 year. At this point, there were significant reductions in RV volumes, RV mass, PR, and RVOT obstruction. It is possible that with longer follow-up the remodeling process would continue and lead to beneficial changes in LV rotational parameters. However, with time, bioprosthetic pulmonary valve function will deteriorate leading to RV adverse remodeling [54]. Hallbergson et al. showed that by 7–10 years after PVR the PR fraction had increased significantly and the RVEDVI had returned to 84% of its pre-PVR value [54]. Thus, the optimal timepoint to assess for improvement in LV rotational parameters is unclear and longitudinal studies post-PVR are needed.

## Conclusions

LV rotation, twist, and torsion in rTOF patients are diminished compared to healthy controls and do not improve at a median of 1 year after PVR despite significant reductions in RV volumes, RV mass, PR, and RVOT obstruction. These findings suggest that factors other than pulmonary valve dysfunction and its sequelae may contribute to LV dysfunction. Additional research is needed to better understand the mechanism of LV rotational abnormalities in rTOF patients and to evaluate whether serial assessment of these parameters can impact prognosis and management decisions.

## Supplementary Information

**Additional file 1: Table S1.** Left ventricular rotational rates and timing of events. **Table S2.** Left ventricular rotational rates and timing of events in repaired tetralogy of Fallot subgroups.

## Data Availability

The dataset generated and/or analyzed during this current study is available upon request.

## Abbreviations

BSA: Body surface area
bSSFP: Balanced steady state free precession
CMR: Cardiovascular magnetic resonance
ECG: Electrocardiogram
FT: Feature tracking
LV: Left ventricle/left ventricular
LVEDVI: Left ventricular end-diastolic volume index
LVEF: Left ventricular ejection fraction
LVESVI: Left ventricular end-systolic volume index
PG: Pressure gradient
PR: Pulmonary regurgitation
PVR: Pulmonary valve replacement
rTOF: Repaired tetralogy of Fallot
RV: Right ventricle/right ventricular
RVEDVI: Right ventricular end-diastolic volume index
RVEF: Right ventricular ejection fraction
RVESVI: Right ventricular end-systolic volume index
RVOT: Right ventricular outflow tract
RVOTO: Right ventricular outflow tract obstruction
TPM: Tissue phase mapping
TOF: Tetralogy of Fallot
TR: Tricuspid regurgitation
