# Supplementary material for: Impact of pulmonary valve replacement on left ventricular rotational mechanics in repaired tetralogy of Fallot
Source: J Cardiovasc Magn Reson. 2021 May 24;23:61. doi: 10.1186/s12968-021-00750-3 (PMC8142485; doi:10.1186/s12968-021-00750-3)
Supplement: Supplementary file 1 — Additional file 1: Table S1. Left ventricular rotational rates and timing of events. Table S2. Left ventricular rotational rates and timing of events in repaired tetralogy of Fallot subgroups. [file 12968_2021_750_MOESM1_ESM.docx]

Table S1 Left ventricular rotational rates and timing of events

|  | **TOF Pre-PVR (n=60)** | **TOF Post-PVR (n=60)** | **Healthy Controls (n=30)** | **TOF Pre-PVR vs. Controls P-value** | **TOF Post-PVR vs. Controls P-value** | **TOF Pre-PVR vs. TOF Post-PVRP-value** |
| --- | --- | --- | --- | --- | --- | --- |
| **Rate of rotation** |  |  |  |  |  |  |
| **Peak systolic apical rotation rate (deg/s)** | 39.7 ± 30.5 | 37.6 ± 30.3 | 53.9 ± 19.5 | **0.009** | **0.003** | 0.661 |
| **Peak diastolic apical rotation rate (deg/s)** | -51.0 ± 42.5 | -49.4 ± 39.9 | -64.6 ± 29.9 | 0.083 | 0.069 | 0.784 |
| **Peak systolic basal rotation rate (deg/s)** | -23.7 ± 18.7 | -20.0 ± 23.6 | -34.9 ± 12.0 | **<0.001** | **<0.001** | 0.312 |
| **Peak diastolic basal rotation rate (deg/s)** | 28.5 ± 30.7 | 22.0 ± 23.6 | 41.8 ± 20.0 | **0.016** | **<0.001** | 0.145 |
| **Peak systolic torsion rate (deg/cm/s)** | 8.9 ± 4.3 | 9.2 ± 4.6 | 12.2 ± 4.3 | **<0.001** | **0.003** | 0.668 |
| **Peak diastolic torsion rate (deg/cm/s)** | -12.1 ± 9.1 | -12.0 ± 7.8 | -15.4 ± 7.4 | 0.084 | **0.047** | 0.930 |
| **Timing of events (% of systole)** |  |  |  |  |  |  |
| **Peak apical rotation (%)** | 91.8 ± 24.3 | 97.9 ± 21.1 | 102.0 ± 16.2 | **0.020** | 0.346 | 0.133 |
| **Peak basal rotation (%)** | 106.7 ± 22.7 | 108.4 ± 18.6 | 111.2 ± 15.6 | 0.271 | 0.484 | 0.491 |
| **Peak apical systolic rotation rate (%)** | 51.4 ± 26.8 | 56.3 ± 27.3 | 46.7 ± 27.3 | 0.446 | 0.122 | 0.280 |
| **Peak apical diastolic rotation rate (%)** | 137.8 ± 21.6 | 141.6 ± 33.5 | 139.9 ± 18.2 | 0.646 | 0.767 | 0.381 |
| **Peak basal systolic rotation rate (%)** | 61.7 ± 27.1 | 65.3 ± 30.6 | 70.8 ± 25.2 | 0.129 | 0.396 | 0.444 |
| **Peak basal diastolic rotation rate (%)** | 163.5 ± 37.5 | 159.7 ± 47.8 | 148.6 ± 16.1 | **0.010** | 0.110 | 0.596 |
| **Peak torsion (%)** | 96.4 ± 24.8 | 104.6 ± 14.2 | 104.9 ± 13.5 | **0.038** | 0.923 | **0.024** |
| **Peak systolic torsion rate (%)** | 56.8 ± 30.6 | 61.7 ± 27.5 | 61.4 ± 26.4 | 0.488 | 0.953 | 0.265 |
| **Peak diastolic torsion rate (%)** | 143.7 ± 23.7 | 140.2 ± 26.8 | 143.0 ± 17.6 | 0.888 | 0.543 | 0.446 |

Values are mean ± standard deviation or N (%). Deg, degrees; TOF, tetralogy of Fallot; and PVR, pulmonary valve replacement.

**Table S2. Left ventricular rotational rates and timing of events in repaired tetralogy of Fallot subgroups**

|  | **Pre-PVR** | | | **Post-PVR** | | | **Pre-PVR vs. Post-PVR** | |
| --- | --- | --- | --- | --- | --- | --- | --- | --- |
| **Variable** | **TOF-PR**  **(n=38)** | **TOF-RVOTO**  **(n=22)** | **P-value** | **TOF-PR**  **(n=38)** | **TOF-RVOTO**  **(n=22)** | **P-value** | **TOF-PR**  **P-value** | **TOF-RVOTO**  **P-value** |
| **Rate of rotation** |  |  |  |  |  |  |  |  |
| **Peak systolic apical rotation rate (deg/s)** | 44.3 ± 34.8 | 31.6 ± 19.5 | 0.074 | 48.9 ± 19.5 | 18.0 ± 35.7 | **<0.001** | 0.477 | **0.046** |
| **Peak diastolic apical rotation rate (deg/s)** | -55.6 ± 45.3 | -43.1 ± 36.7 | 0.275 | -57.7 ± 28.0 | -35.2 ± 52.5 | 0.074 | 0.791 | 0.361 |
| **Peak systolic basal rotation rate (deg/s)** | -24.5 ± 18.8 | -22.3 ± 19.0 | 0.664 | -20.2 ± 21.9 | -19.8 ± 26.8 | 0.951 | 0.328 | 0.697 |
| **Peak diastolic basal rotation rate (deg/s)** | 29.5 ± 34.3 | 26.9 ± 24.0 | 0.758 | 24.1 ± 21.7 | 18.4 ± 26.8 | 0.371 | 0.332 | 0.279 |
| **Peak systolic torsion rate (deg/cm/s)** | 9.4 ± 4.2 | 7.9 ± 4.4 | 0.197 | 9.9 ± 3.8 | 8.0 ± 5.5 | 0.162 | 0.624 | 0.961 |
| **Peak diastolic torsion rate (deg/cm/s)** | -12.4 ± 9.8 | -11.5 ± 7.7 | 0.732 | -12.6 ± 5.1 | -10.9 ± 11.0 | 0.497 | 0.901 | 0.722 |
| **Timing of events (% of systole)** |  |  |  |  |  |  |  |  |
| **Peak apical rotation (%)** | 96.9 ± 24.1 | 83.0 ± 22.5 | **0.033** | 95.8 ± 24.6 | 101.4 ± 13.0 | 0.251 | 0.837 | **0.003** |
| **Peak basal rotation (%)** | 109.6 ± 19.5 | 101.7 ± 27.2 | 0.195 | 112.3 ± 16.2 | 101.8 ± 21.0 | **0.036** | 0.419 | 0.968 |
| **Peak apical systolic rotation rate (%)** | 57.0 ± 29.1 | 41.6 ± 19.4 | **0.032** | 56.1 ± 24.4 | 56.6 ± 32.3 | 0.945 | 0.871 | 0.058 |
| **Peak apical diastolic rotation rate (%)** | 138.8 ± 17.4 | 136.1 ± 27.8 | 0.689 | 133.4 ± 16.1 | 155.6 ± 48.7 | **0.049** | 0.187 | **0.030** |
| **Peak basal systolic rotation rate (%)** | 64.9 ± 27.5 | 56.2 ± 26.0 | 0.231 | 71.4 ± 29.9 | 54.7 ± 29.7 | **0.041** | 0.291 | 0.837 |
| **Peak basal diastolic rotation rate (%)** | 160.9 ± 36.1 | 168.1 ± 40.2 | 0.476 | 150.2 ± 28.7 | 176.0 ± 67.3 | 0.100 | 0.093 | 0.636 |
| **Peak torsion (%)** | 103.8 ± 22.9 | 83.7 ± 23.3 | **0.002** | 104.2 ± 16.1 | 105.4 ± 10.3 | 0.724 | 0.925 | **<0.001** |
| **Peak systolic torsion rate (%)** | 62.0 ± 29.5 | 47.9 ± 30.9 | 0.084 | 67.1 ± 28.2 | 52.4 ± 23.9 | **0.045** | 0.377 | 0.511 |
| **Peak diastolic torsion rate (%)** | 145.6 ± 24.0 | 140.5 ± 23.4 | 0.431 | 135.9 ± 16.7 | 147.5 ± 37.9 | 0.187 | **0.027** | 0.505 |

Values are mean ± standard deviation. TOF-PR are repaired tetralogy of Fallot patients with a pulmonary regurgitation fraction >40% and a right ventricular outflow tract peak gradient ≤25 mm Hg. TOF-RVOTO are rTOF patients with a PR fraction ≤30% and a RVOT peak gradient or tricuspid regurgitation peak gradient ≥40 mm Hg. PR, pulmonary regurgitation; PVR, pulmonary valve replacement; RVOTO, right ventricular outflow tract obstruction; TOF, tetralogy of Fallot.
